# An Account of Diseases in an Eastern District of London, from August 20, to September 20, 1808

**Published:** 1808-10

**Authors:** 


### *An Account of Diseases in an Eastern District of London, from August 20, to September 20,* 1808.

| | | | |
|---|---|---|---|
| ACUTE DISEASES. | | Rheumatismus Acutus | 3 |
| Typhus - - - | 2 | CHRONIC DISEASES. | |
| Hæmoptysis - - | 3 | Catarrhus - - - | 4 |
| Cholera - - - | 12 | Tussis - - - | 8 |

Tussis

| | |
|---|---|
| Tussis cum Dyspnœa | 6 |
| Phthisis Pulmonalis | 3 |
| Hydrothorax - - | 2 |
| Pleurodyne - - | 4 |
| Ascites - - - | 3 |
| Anasarca - - | 5 |
| Gastrodynia - - | 4 |
| Hemiplegia - - | 2 |
| Dyspepsia - - | 4 |
| Hypochondriasis - | 2 |
| Menorrhagia - - | 4 |
| Fluor Albus - - | 5 |
| Amenorrhœa - - | 3 |
| Rheumatismus Chronicus | 7 |
| PUERPERAL DISEASES. | |
| Menorrhagia Lochialis | 3 |
| Dolores post Partum | 5 |
| Mastodynia - - | 4 |
| Diarrhœa - - | 9 |
| Enterodynia - - | 7 |
| INFANTILE DISEASES. | |
| Aphthæ - - - | 3 |
| Erysipelas Infantile | 1 |
| Ophthalmia - - | 3 |
| Hydrocephalus - | 2 |
| Dentitio - - - | 3 |

In a former Report, a few instances of Cholera were taken notice of, which, it was remarked, had occurred at a period much earlier than that in which this disease usually makes its appearance. The very uncommon heat of the weather was sufficient to account for this, and accordingly, upon a change in the temperature of the atmosphere, this complaint gradually subsided.

It has been observed, indeed, that in hot climates, if, after an excess of heat and dryness, a considerable quantity of rain falls, by which the atmosphere is suddenly cooled, this disease prevails to a considerable degree. The sudden check to perspiration which may be given at such a time, may have some influence in producing or promoting the complaint. The number of patients labouring under this disease has lately increased.

Amongst the cases referred to in the list there were some that were attended with large discharges of a bilious kind from the stomach and bowels. In many instances of intestinal evacuation, the account given by the patient, is not to be received without particular attention and inquiry, as they are apt to be alarmed by the frequent calls to go to stool; and to suppose that the discharges are abundantly more copious than they really are: but in the cholera, the report is generally well founded, though to persons unacquainted with the disease, it would hardly appear credible that so large a quantity could be discharged.

This disease seems to be produced either by a change in the quantity, or quality, of the bile which is secreted, and afterwards poured into the alimentary canal. If, by the state of the atmosphere, or any other occasional cause, this irregular secretion takes place, very copious discharges of bile are immediately produced both from the stomach and intestines, intestines. These are often accompanied by a spasmodic affection of the bowels, which also extends itself to the lower extremities.

By these copious evacuations and the pain produced by the spasm, the patients strength is sometimes suddenly reduced, and he soon falls a victim to the disease: but in other instances, the complaint after a few days gradually subsides. Weak diluting liquors taken frequently, and in as large a quantity, as the stomach will bear, without exciting vomiting, answer the purpose of correcting the bile, and carrying it gently out of the system, whilst opiates, by moderating the action of the liver, and diminishing its secretions, and at the same time counteracting the disposition to spasm, serve to bring the disease to a favourable crisis.

